# Efficient on-chip platform for coherent light-matter coupling using bound states in the continuum

**DOI:** 10.1126/sciadv.adu0976

**Published:** 2025-04-25

**Authors:** Pai Zhou, Hui-Zhen Zhang, Tingmei Li, Zhong-Shan Zhang, Yu-Hui Chen, Xiangdong Zhang

**Affiliations:** ^1^ Key Laboratory of advanced optoelectronic quantum architecture and measurement of Ministry of Education, Beijing Key Laboratory of Nanophotonics & Ultrafine Optoelectronic Systems, School of Physics, Beijing Institute of Technology, Beijing 100081, China.; ^2^ Institute of Physics, Chinese Academy of Sciences, P.O. Box 603, Beijing 100190, China.

## Abstract

Storing and retrieving photonic qubits are key functionalities in future optical quantum networks, and integrating scalable optical-memory units is crucial as these networks expand. However, attempts to combine silicon photonics and erbium ions for telecom memories, without losing the scalable and low-loss properties of silicon chips, face challenges because of limited light-matter interactions and potential extra decoherence. Here, we present an efficient silicon-chip platform using bound states in the continuum to overcome these limitations. In addition to a low propagation loss of 0.5 ± 0.5 decibels per centimeter, our experiments demonstrate an order-of-magnitude enhancement in light absorption compared to previous traditional silicon hybrid designs. Using these properties, we demonstrated photon echoes in our waveguide structures, revealing a coherence time of 2.6 ± 0.6 microseconds at zero magnetic field, closely matching that of bulk crystals. These characteristics make the bound state in the continuum platform a promising candidate for realizing integrated optical memories for quantum network applications.

## Teaser

An efficient on-chip platform via bound states in the continuum combines silicon photonics with erbium ions for quantum memories.

## INTRODUCTION

Optical quantum memories, acting as storage units for the fragile quantum information carried by photons, are fundamental building blocks for scalable quantum networks. These memories facilitate the realization of exciting applications such as distributed quantum computing and sensing (*1*–*3*). However, as the networks expand, scaling these memories to meet the increasing demand of quantum resources becomes crucial.

Integrated quantum memory, with its potential for seamless integration with existing on-chip devices, provides a promising pathway for achieving the required scalability (*4*, *5*). While a range of materials for integrated memories are being explored, rare-earth ion–doped solid-state materials emerge as a leading platform (*6*–*8*) because of their exceptional properties: long coherence times (*9*–*11*), high storage efficiency (*12*, *13*), and multimode capabilities (*8*, *14*). Initial efforts focused on direct fabrication within crystals using techniques such as ion diffusion (*15*, *16*), focused ion-beam milling (*17*, *18*), and femtosecond laser writing (*14*, *19*–*21*). However, the scalability of these micro quantum memories is restricted by the ongoing development of fabrication techniques tailored to specific materials.

Another method toward on-chip quantum memory involves integrating silicon photonic structures with bulk rare earth–doped crystals, particularly erbium-doped crystals that emit at 1.5 μm. This hybrid approach, while ideal for its compatibility with advanced silicon fabrication techniques and the existing optical telecom infrastructure, faces a fundamental limitation. The high refractive index of silicon confines most of the optical field within the silicon material (*22*–*25*), which means that only the erbium ions located near the material interfaces can effectively interact with the optical field. This not only results in weak light-matter interactions but also causes extra decoherence, as ions near the interface experience a nonuniform charge environment. While using small mode-volume resonators shows promise in enhancing light-matter interactions (*25*, *26*), it brings more ions to the material interfaces and further increases the likelihood of decoherence (*8*). Consequently, enhancing memory scalability through silicon photonics often sacrifices key performance metrics such as light-matter interaction strength and coherence time.

On the other hand, recent advancements in bound state in the continuum (BIC) photonics offer distinct solutions for integrated optics. The concept of BICs, first introduced by von Neumann and Wigner in 1929 (*27*), describes a phenomenon where a confined mode (bound state) coexists with leaky environment modes (the continuum) (*28*, *29*). Photonics BICs (*30*–*34*) manifest as a spatially confined optical mode existing within a leaky background, decoupled to surrounding modes. Integrating BIC structures into hybrid silicon photonics holds substantial potential for enhancing on-chip memory properties, but its feasibility remains an open question (*35*–*38*).

Here, we demonstrate an efficient on-chip optical-memory platform that is enabled by BICs within a hybrid structure of silicon and erbium-doped crystal. Compared to conventional silicon waveguide–based hybrid structures, our BIC structure effectively confines light while minimizing radiative loss, leading to a notable reduction in propagation loss and enhancement in the light absorption of the erbium ions. In addition, photon echo measurements indicate that the coherence time of the erbium ions shows no degradation compared to that of bulk materials at zero magnetic field. This BIC structure, readily mass manufacturable using silicon fabrication techniques, is promising for the development of practical, scalable quantum memory chips.

## RESULTS

### Design of the BIC structure

**Fig. 1.**
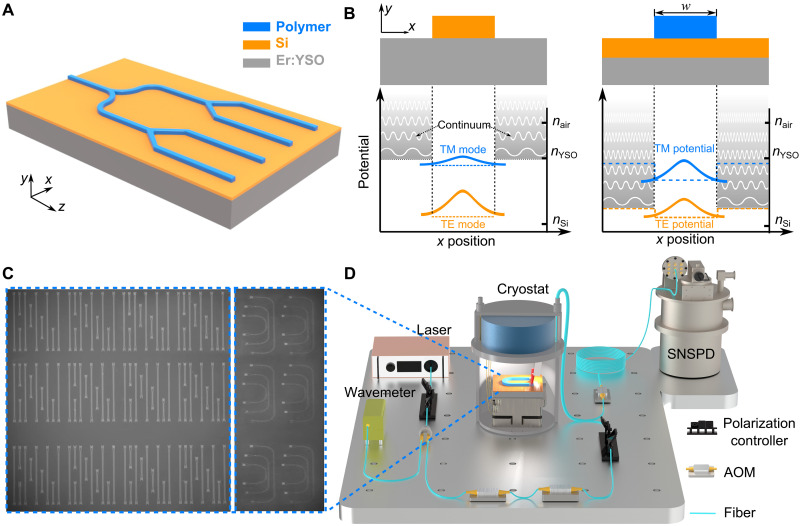
Illustration of the BIC structure and experimental setup. (**A**) Schematic of the BIC structure for integrated photonic quantum memories, comprising a Er:YSO crystal substrate (gray), a thin film of silicon (orange), and a patterned polymer layer (blue). (**B**) Conceptual illustration of the construction of BIC waveguides. Left panel: cross section of a traditional silicon waveguide patterned on top of a YSO crystal and its effective potentials for both TE-like and TM-like modes as noted. The effective indices of the fundamental TE and TM modes for a silicon waveguide (200 nm by 600 nm) on top of a YSO substrate are 2.48 and 1.80, respectively. Right panel: cross section of our BIC waveguide and its corresponding potentials for both TE and TM modes as noted. The effective index of the TM mode of our BIC structure is 2.07, while the maximum effective index of the continuum TE modes is 2.72. The positions corresponding to the refractive indices of air, YSO crystal, and silicon are marked on the right of these potential diagrams. (**C**) Microscope images of the BIC optical memory chip. The regions of straight and curved waveguides are spliced together for visibility. Bright regions at the waveguide ends correspond to input/output grating couplers. (**D**) Schematic of our experimental setup. AOM, acoustic optical modulator.

Figure 1A illustrates the design of our BIC structure, which is composed of an erbium-doped yttrium orthosilicate crystal (Er:YSO) substrate, a silicon thin film, and a patterned polymer layer. The refractive indices of these materials at 1.5 μm are 1.78, 3.37, and 1.54, respectively (*39*). Using the concept of effective potential, the representation of our structure as a BIC is shown in Fig. 1B. An optical mode ϕ(**r**) propagating through a waveguide along the *z*-direction can be described by (*35*, *36*)

$$i2\beta \frac{\partial }{\partial z}\phi \left(r\right)=\left[-\nabla^{2}+U\left(r\right)\right]\phi \left(r\right)$$

where β is the propagation constant, *n*(**r**) is the material refractive index, *k* = 2π/λ is the vacuum wave number, and $U\left(r\right)=\beta^{2}-n^{2}\left(r\right)k^{2}$ is the effective potential. This equation is similar to the Schrödinger equation, with time-dependent evolution replaced by the spatial-*z* evolution.

Traditional waveguides, such as a silicon waveguide on top of a YSO crystal as shown in Fig. 1B, confine light primarily through index guiding. In these multilayer structures, both transverse electric (TE) and transverse magnetic (TM) propagating modes are supported. The effective refractive indices of the TE and TM modes are between *n*_Si_ and *n*_YSO_. Typically, the TM mode has a lower index than the TE mode, indicating a higher position in the effective potential diagram (Fig. 1B). In the surrounding regions, the infinite YSO substrate supports extended modes with effective indices lower than *n*_YSO_. Taking a standard silicon waveguide (200 nm by 600 nm) as an example, the effective indices of the fundamental TE and TM modes are 2.48 and 1.80, respectively. The notable index difference between the TE mode and the YSO substrate, translating to a substantial potential barrier, results in light confinement and weak coupling to the continuum modes. Consequently, traditional silicon waveguides show low radiative propagation losses, typically exceeding 6 dB/cm for amorphous silicon waveguides (*40*) and 0.1 dB/cm for crystalline silicon waveguides (*41*–*43*).

The light confinement mechanism in our BIC structure fundamentally differs from that of conventional waveguides. The right panel of Fig. 1B illustrates the fundamental TM and TE modes in different regimes of our structure. Because of the infinite extent, the regimes surrounding the polymer support a continuum of TE and TM modes. These continuum modes are of higher energies than their corresponding fundamental modes. Introducing a polymer layer locally increases the effective indices of both TM and TE modes. Therefore, the bound TM mode within the polymer region shifts downward in the potential diagram, residing energetically far from the TM continuum outside the polymer. However, this bound TM mode coexists with the TE continuum, as illustrated in the right panel of Fig. 1B. There, the effective index of the bound TM mode (*n*_eff,TM_ = 2.07) is lower than the maximum index of the TE continuum modes (*n*_continuum_ = 2.72). Conventional waveguide theory predicts that no guided modes should exist under this condition, because the coupling to the surrounding continuum modes arises at the polymer boundaries and leads to substantial radiative losses, preventing the structure from effectively guiding light.

However, by precisely adjusting the polymer width, we can achieve destructive interference of the scattered waves, effectively canceling the coupling between the bound mode and the leaky continuum. This condition is known as an optical BIC, which results in near-zero radiative loss and strong light confinement despite having a lower effective index than its surrounding. This BIC effect releases the constraint that the effective index of a guiding mode must be larger than that of its continuum, offering a fundamentally different approach to light confinement.

On the basis of the above model, we designed and fabricated (see Materials and Methods) a series of waveguide samples with varying lengths corresponding to different polymer widths *w.* The scanning electron microscope images of our on-chip samples are shown in Fig. 1C (detailed in section S1). Polarization-sensitive grating couplers (refer to section S2) were integrated at both ends of the polymer waveguide to couple light into and out of the chip. As depicted in the experimental setup of Fig. 1D, our samples were cooled to 4 K using a homemade cryostat. Light was delivered through a fiber array (8 by 1) (or fiber tips) and detected by a superconducting nanowire single-photon detector (SNSPD).

### Observation of the BIC effect

**Fig. 2.**
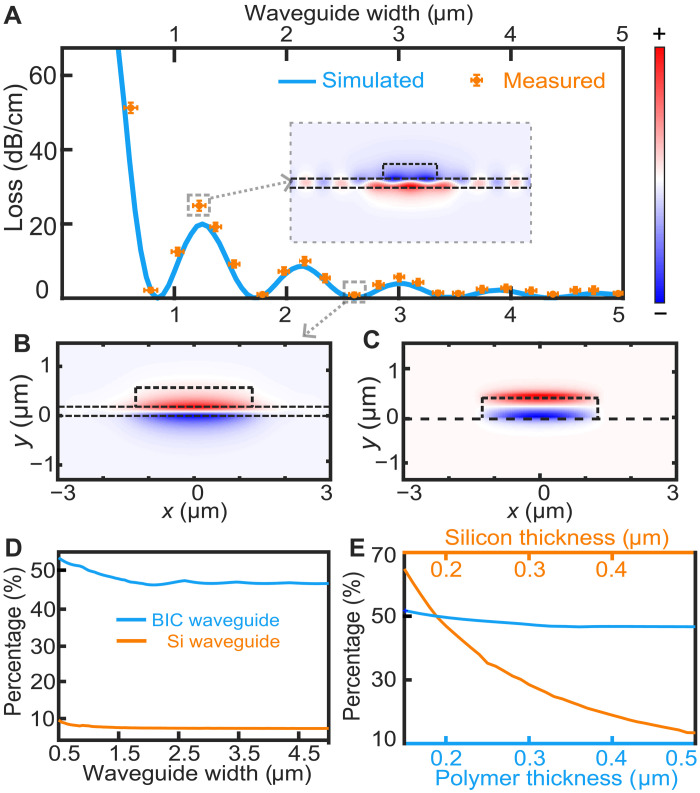
Demonstration of the BIC effect. (**A**) Simulated and measured propagation loss of the BIC waveguides as a function of polymer width *w*. Inset: optical field distribution of the electric component *E*_*z*_ corresponding to the marked high-loss point. (**B**) Optical field distribution (*E*_*z*_) corresponding to the marked low-loss point, where the width *w* = 2.6 μm. (**C**) Optical field distribution (*E*_*z*_) of a silicon waveguide on top of a YSO substrate. The silicon waveguide has a thickness of 350 nm and a width of 2.6 μm. (**D**) Ratio of optical energy confined in the YSO crystal to the total energy as a function of waveguide width for BIC (blue) and silicon (orange) waveguides. (**E**) Analysis of the ratio of optical energy confined in the YSO crystal to the total energy in our BIC waveguide. Blue, ratio for varying polymer thickness; orange, ratio for varying silicon layer thickness.

We used COMSOL Multiphysics software to simulate the propagation loss of our designed hybrid waveguide structures (see section S3). The blue line in Fig. 2A represents the propagation loss of the bound TM mode in a straight waveguide for different widths *w*. The loss exhibits peaks at certain geometric parameters while substantially decreasing at others, averaging around 10 dB/cm. For the point *w* = 1.2 μm, which corresponds to a high propagation loss of 20 dB/cm, the inset in Fig. 2A clearly shows a strong interaction between the bound mode and the extended modes on both sides. This interaction implies the scattering of the TM mode into the continuum at the polymer boundaries, leading to a high propagation loss at this specific width.

However, as shown in Fig. 2B, at a distinct BIC point with a polymer width of *w* = 2.6 μm, the field distribution reveals that the strong coupling between the bound mode and the TE continuum observed in Fig. 2A is no longer present. At this BIC point, the propagation loss can be extremely low, with a simulated value as minimal as 0.02 dB/cm. Moreover, our calculations suggest that the tolerance in waveguide width required to maintain a loss rate below 1 dB/cm can be as large as 200 nm (section S3), which can be reliably achieved with current microfabrication techniques.

Measurements of input and output light intensity across these samples allowed us to deduce the propagation loss for each width (see section S4). The results, shown in Fig. 2A, align well with our calculations. For the aforementioned waveguide with *w* = 2.6 μm with a loss of 0.02 dB/cm in simulations, the measured propagation loss was 0.5 ± 0.5 dB/cm. This measurement was limited by the reliably achievable waveguide length with our lithography system, restricting our ability to measure intensity differences of ~0.5%. Despite this limitation, the measured loss is notably better than that of commercially available amorphous silicon waveguides [typically ~6 dB/cm (*40*)]. Further reduction in propagation loss will likely require both more precise measurement techniques and the use of crystalline silicon instead of amorphous silicon. In our simulations and experiments, BIC points are identified for waveguide widths of 1.7, 2.6, 3.5, and 4.5 μm.

Beyond exhibiting low propagation loss, our BIC structure outperforms conventional silicon waveguide–based hybrid structures by enabling flexible control of the spatial field distribution. Traditional waveguides primarily confine light within high-refractive-index materials. Figure 2C shows the field distribution of a traditional silicon waveguide on a YSO crystal, with dimensions of 2.6 μm in width and 350 nm in height. The results demonstrate that most of the optical field is concentrated within the silicon, with only 8% penetrating into the YSO crystal, which fundamentally limits the low absorption of light in this kind of structure (see section S5).

In contrast, the field distribution shown in Fig. 2B reveals that our BIC structure allows a substantial portion of the optical field to penetrate into the YSO crystal. Our calculations indicate that more than 45% of the optical field can penetrate the YSO crystal when the waveguide width varies from 0.5 to 2 μm, as shown in Fig. 2D. In particular, the effective penetration of the field exhibits a weak dependence on the thickness of the polymer layer, as shown in Fig. 2E. This behavior is attributed to the exponential decay of the optical field within the polymer. As long as the thickness exceeds this decay length, variations in polymer thickness have a minimal impact. This favorable tolerance in our BIC structure indicates that precise control over the polymer thickness is not critical. The thickness of the silicon layer, however, plays a notable role in the performance of the BIC waveguide. As the thickness of silicon increases to 0.5 μm, the characteristics of the BIC become less pronounced with the penetration decreasing to only 12%.

### Coupling of the optical field and erbium ions

**Fig. 3.**
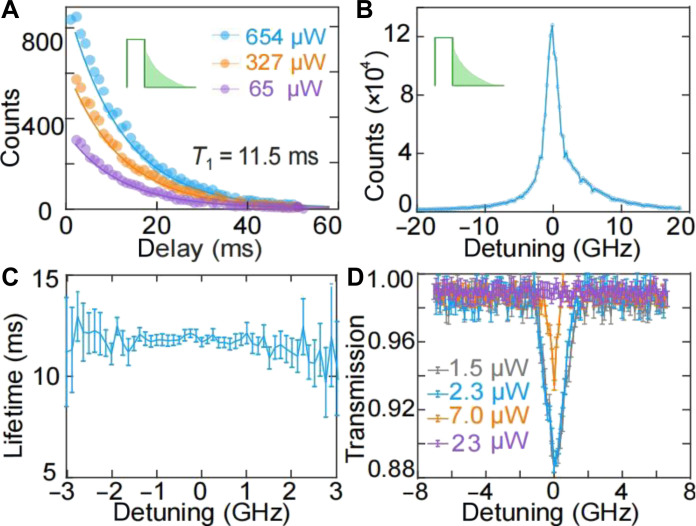
Coupling between erbium ions and the BIC waveguide. (**A**) Photoluminescence intensity measured at different delays after pulsed excitation. The duration of the excitation pulse was 20 ms, and the corresponding power is varied as indicated. The integration detection time for the SNSPD is 1 ms. Points, experimental data; solid lines, fitted curves. (**B**) Photoluminescence intensity measured after pulsed excitation as a function of detuned frequency of the laser. The power of the excitation pulses is 654 μW, while the SNSPD integration time was increased to 50 ms. The linewidth is 2.1 GHz. (**C**) Measured excited-state lifetime of erbium ions for varying laser detunings. (**D**) Continuous-wave absorption measurements of the BIC structure. Notable saturation of the inhomogeneous line occurs at the input power of 23 μW, with negligible saturation below 2.3 μW. The inhomogeneous linewidth measured at 2.3 μW is 1.6 GHz.

The high penetration rate into the YSO crystal directly indicates that the light field interacts with the erbium ions more efficiently. To experimentally confirm this enhanced interaction, we applied a 20-ms resonant pulse at 1536.47 nm to excite the erbium ions (*44*, *45*), followed by measuring the photon luminescence (PL) signal using an SNSPD (detailed in Materials and Methods and sections S6 and S7). After excitation, the erbium ions release energy by emitting photons. By detecting the PL signal at different postexcitation delays, we can determine the exited-state lifetime of the erbium ions. Figure 3A presents the measured PL signal as a function of time delays for different pump powers. Fitting of these data yielded an excited-state lifetime of 11.5 ms.

To further substantiate that the PL emission originates specifically from the erbium ions, we varied the frequencies of our excitation laser in our PL measurements (see section S8). When the laser frequency was outside the absorption band of the erbium ions, no PL signals were observed. By adjusting the laser frequency, we detected the PL signal within a range characterized by a full width at half maximum of 2.1 GHz, as shown in Fig. 3B. Throughout this frequency range, all measurements consistently indicated that erbium ions have a lifetime of *T*_1_ = 11.5 ± 0.2 ms (Fig. 3C).

After establishing the coupling between the optical field and the erbium ions, we focused on demonstrating that this coupling is enhanced. We used a continuous-wave laser to measure the absorption spectrum of a 1.5-mm BIC waveguide. Figure 3D shows the absorption spectra for different input laser powers. At an input power of 23 μW, the absorption signal is barely noticeable, which is attributed to the long excited-state lifetime of erbium ions, leading to easy saturation under continuous-wave excitation. In contrast, decreasing the input power below 2.3 μW results in a peak absorption of 11.5%, along with an inhomogeneous linewidth of 1.5 GHz. This absorption, measured over a waveguide length of 1.5 mm (limited by the optimal fabrication area of our electron beam lithography system), corresponds to an absorption loss of 3.6 dB/cm (or an absorption coefficient of 0.8 cm^−1^), representing an order-of-magnitude enhancement compared to the absorption coefficient reported by Craiciu *et al.* (*22*), where a conventional slab silicon was placed on top of an Er:YSO crystal. The absorption coefficient in the literature is deduced by comparing the *Q*-factors of their F-P cavity with and without erbium absorption. Note that the observed absorption enhancement depends on the mode characters of the reference silicon waveguide. However, for a standard silicon waveguide with dimensions of 200 nm by 600 nm, the TM-like mode has a refractive index of 1.80. Compared to the YSO substrate (*n* = 1.78), such a small index contrast is not suitable for guiding light. For the TE guided mode in standard silicon waveguides, our BIC structure provides a consistent ~20-fold enhancement (see section S3). Furthermore, compared to the absorption of 2.5 cm^−1^ of our reference bulk Er:YSO crystal, this measured value of 0.8 cm^−1^ of our BIC waveguide is ~32% of the bulk crystal absorption, aligning with the simulated 45% field penetration in the YSO crystal (see section S9).

Note that the inhomogeneous linewidth obtained from the PL measurements (Fig. 3C) is different from that of the absorption measurements (Fig. 3D). This discrepancy stems from the long excited-state lifetime of the erbium ions, making it easier to excite the ions across the entire gigahertz inhomogeneous linewidth with moderate laser power. In contrast, the inhomogeneous linewidth observed in the BIC waveguide is consistent with the 1.6-GHz linewidth measured in our bulk Er:YSO absorption experiments at 1536.47 nm (detailed in section S8). These findings suggest that the inhomogeneous linewidth remains unchanged during chip fabrication.

### Photon echo measurements

**Fig. 4.**
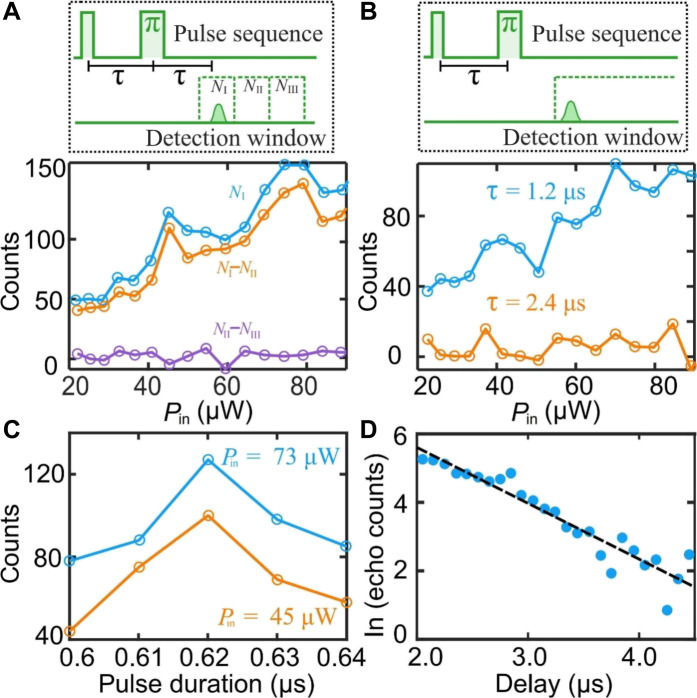
Photon echo measurements. (**A**) Two-pulse photon echo. Inset: top: illustration of the pulse sequence applied to the chip and the detection windows. The π/2 and π pulses have durations of 0.31 and 0.62 μs, respectively, with a wait time τ = 0.6 μs. Photon counts *N*_I_, *N*_II_, and *N*_III_ are integrated over three successive 1-μs detection windows (I, II, and III). Detection window I opens 0.2 μs after the π pulse. Bottom: photon counts in window I and the photon number difference between different windows as a function of input laser power. Blue, *N*_I_; orange, *N*_I_-*N*_II_; purple, *N*_II_-*N*_III_. (**B**) Comparison of the echo emissions for two different wait times τ. Top: schematic of the pulse sequence and detection windows. Bottom: echo signal as a function of input power for wait time τ = 1.2 μs (blue) and τ = 2.4 μs (orange). The SNSPD integration time is 20 μs. (**C**) Photon echo for varying width of the second pulse. Blue, for an input laser power of 73 μW; orange, for 45 μW. (**D**) Exponential decay of the photon echo. The determined optical coherence time is *T*_2_ = 2.6 ± 0.5 μs. Points, experimental data; dashed line, fitted curve. The input laser power is 75 μW.

Besides the inhomogeneous linewidth, our BIC structure also demonstrates no degradation of the homogeneous linewidths of erbium ions, a crucial requirement for optical quantum memory applications. We conducted two-pulse photon echo experiments to investigate the homogeneous linewidth. The applied pulse sequence is illustrated at the top of Fig. 4A. A π/2 pulse with a duration of 0.31 μs is input to the waveguide, followed by a wait time τ, after which a π pulse with a 0.62-μs duration is applied to rephase the atomic ensemble. This sequence is expected to generate a photon echo at the same wait time τ. Setting τ = 0.6 μs, we used an SNSPD to measure the integrated photon number within a 1-μs integration window (window I in Fig. 4A), which was set to capture the expected echo signal. The emission signal shown by the blue line in Fig. 4A reveals that the emission increases with increasing laser power.

We distinguished this photon echo emission from spontaneous emission by comparing photon counts in different time windows. Specifically, we further recorded photon counts in 1-μs windows II and III, immediately following window I (Fig. 4A). Given its 11-ms excited-state lifetime, spontaneous emission from the erbium ions would be present in all the three detection windows and exhibits at most a 10^−4^ difference between successive windows. However, the photon count difference *N*_I_ − *N*_II_ between windows I and II (orange line in Fig. 4A) was notably larger, whereas the photon count difference in windows II and III (purple line in Fig. 4A) approached zero. These contrasting trends confirm that the detected signal in window I is primarily from coherent photon echo, and the small remaining photon counts in windows II and III are attributed to spontaneous emission and/or unblocked light.

We further investigated the relationship between echo emission and the wait time τ. We measured the photon numbers after the π pulse using a 20-μs integration window, as illustrated in Fig. 4B. A longer integration window than that used in Fig. 4A was chosen to account for possible changes in the echo’s temporal shape with varying laser power. When τ = 1.2 μs, the detected photon counts exhibited a similar dependence on input laser power as observed in Fig. 4A. However, a substantial reduction in signal strength was observed at τ = 2.4 μs (Fig. 4B), which shows the expected decrease in echo strength with increasing τ. This τ dependence suggests that the detected signal originates from a coherent two-pulse photon echo.

Determining the optimal π pulse duration for echo generation in our BIC waveguide using the conventional PL method (*26*) faces several challenges. These include the inhomogeneous distribution of the optical field, the susceptibility of erbium ions to saturation, and the presence of background spontaneous emission. In addition, achieving a high signal-to-noise ratio with the SNSPD while maintaining a nanosecond time resolution proves difficult. Alternatively, we fixed the input power and varied the pulse duration in the echo measurements to optimize the π pulse duration. As shown in Fig. 4C, the strongest echo signal occurs at a pulse duration of 0.62 μs for a laser input of 42 μW. Using these experimental parameters, Fig. 4D measures the maximum intensity of the photon echo as a function of the delay τ. The results suggest that the optical coherence time of the erbium ions is *T*_2_ = 2.6 ± 0.6 μs, which is consistent with the 3.7-μs coherence time measured in the bulk material with a similar concentration of erbium of 32 parts per million at a lower temperature of 1.5 K (*46*, *47*). At zero field, the optical coherence is short. Under these conditions, we do not observe a further degradation of coherence because of the fabrication of our BIC structure.

## DISCUSSION

In conventional silicon waveguide–based hybrid structures, the nonuniform charge environment near the interface hinders the preservation of the erbium coherence properties. For example, using focused ion-beam techniques to make microstructures in Er:YSO crystals results in a 4-GHz shift in the 1.5-μm optical transition of erbium ions, as well as an increase in the inhomogeneous linewidth from 1.2 to 5 GHz (*23*). This degradation in properties is commonly observed in various types of integrated waveguide memories (*48*). Our measurements indicate that both the inhomogeneous and homogeneous linewidths of erbium ions in our BIC structure are largely consistent with those of bulk material at zero magnetic field (*18*, *49*). While this result suggests coherence preservation within the limits of our current experimental conditions, longer-timescale investigations are needed to assess potential coherence reductions. Specifically, studies on rare earth–doped nanobeam devices demonstrate coherence comparable to bulk material at the microsecond scale but exhibit reductions around 100 μs, likely caused by heating and/or the focused ion-beam fabrication process. Therefore, future research will focus on refining our measurement techniques to investigate coherence over longer timescales and to further characterize the effects of fabrication on coherence, building upon the efficient coupling we have achieved here.

The advantages in our BIC structure highlight its promising potential for quantum network applications. With the proven improvements in light-matter interaction and the preservation of erbium’s coherence properties, the BIC platform can readily use quantum memory protocols currently used in bulk materials. For practical quantum network applications, achieving high efficiency is particularly crucial for an on-chip multinode system. It is noteworthy that our Er:YSO has an inhomogeneous linewidth of 1.6 GHz, whereas a high-quality YSO crystal can demonstrate a much narrower linewidth of 0.2 GHz (*6*, *44*, *50*). This indicates that the use of a better-quality crystal could increase the absorption coefficient of bulk material to 8 cm^−1^. Under such conditions, achieving 90% absorption would require only a BIC waveguide length of 0.3 cm. This efficient on-chip optical memory facilitates the realization of multiplexed information processing in a single optical chip. Moreover, the ability of the BIC structure to be mass manufactured is a major step toward the development of practical quantum networks. These BIC structures are compatible with conventional silicon thin-film technology, enabling the fabrication of tens of waveguides on a single chip through established routing processes, as demonstrated in Fig. 1C. Experimental testing of multiple waveguides within an area of 4 by 4 mm on the same chip revealed consistent optical properties of erbium ions (section S10), highlighting the homogeneity of the BIC device.

In conclusion, we have demonstrated a scalable optical-memory platform using BIC within a hybrid structure of silicon photonics and erbium-doped crystals, which overcomes a critical limitation of conventional hybrid silicon approaches: the trade-off between strong light-matter interaction and scalability. Our BIC platform achieves not only a low propagation loss of 0.5 dB/cm but also a substantial enhancement in the absorption coefficient. The homogeneous and inhomogeneous linewidths of the erbium ions show no degradation under our experimental conditions. Further enhancement in storage efficiency is possible by extending the interaction length or using high-quality crystals. The observed enhanced absorption, combined with the BIC’s inherent advantages of scalability and minimal loss, shows notable promise for a wide range of applications in the development of large-scale quantum networks.

## MATERIALS AND METHODS

### Sample fabrication

The samples were constructed on an Er:YSO crystal doped with 38–parts per million erbium ions. A 200-nm amorphous silicon layer was deposited on the Er:YSO substrate through plasma-enhanced chemical vapor deposition, followed by a spin-coated 350-nm layer of polymethyl methacrylate. Waveguides and grating couplers were then patterned on the polymer layer using electron beam lithography.

### Experimental measurements

The refractive index of the silicon thin film was measured using an ellipsometer. The experimental setup, designed for testing optical chips, consists of four main components: a laser source, a pulse control system, a cryostat, and a single-photon superconducting nanowire detector. Three acousto-optic modulators were used to gate the laser input pulses and control the timing of photon detection. Samples with varying polymer widths *w* and waveguide lengths were fabricated on a single chip, characterized at room temperature, and subsequently cooled to 4 K using a homemade cryostat.

### Straight and U-shaped waveguides

Optical chips containing multiple straight waveguides were used to measure the propagation losses of waveguides of different widths. Each chip included 300 straight waveguides, with 10 of different lengths (within a region of 1 mm by 1 mm) for each width. For the photon echo measurements, we fabricated multiple U-shaped waveguides, each 1.5 mm long, within an area of 4 mm by 4 mm on another chip.

## Supplementary Materials

### This PDF file includes:

Sections S1 to S10 Figs. S1 to S15
