# Supplementary material for: Efficient on-chip platform for coherent light-matter coupling using bound states in the continuum
Source: Sci Adv. 2025 Apr 25;11(17):eadu0976. doi: 10.1126/sciadv.adu0976 (PMC12024644; doi:10.1126/sciadv.adu0976)
Supplement: Supplementary file 1 — Sections S1 to S10 Figs. S1 to S15 [file sciadv.adu0976_sm.pdf]

Science Advances
AAAS

# Supplementary Materials for

## Efficient on-chip platform for coherent light-matter coupling using bound states in the continuum

Pai Zhou *et al.*

Corresponding author: Yu-Hui Chen, stephen.chen@bit.edu.cn; Xiangdong Zhang, zhangxd@bit.edu.cn

*Sci. Adv.* **11**, eadu0976 (2025)
DOI: 10.1126/sciadv.adu0976

**This PDF file includes:**

Sections S1 to S10
Figs. S1 to S15

# I. SAMPLE FABRICATIONS

The fabrication process of our sample is illustrated in Fig. S1**a**. We used a 1-mm thick yttrium orthosilicate (YSO) crystal, doped with 38-ppm concentration of erbium ions, as the substrate. A 200-nm thin film of amphourous silicon was then deposited onto the YSO substrate using plasma enhanced chemical vapor deposition. Subsequently, a layer of polymethyl methacrylate (PMMA950A5), functioning as an electron beam resist, was spin-coated onto the silicon film at a rotational speed of 3500 rounds per minute, resulting in a film thickness of 350 nm. Waveguides and grating couplers were then patterned on the polymer layer using electron beam lithography. Note that this fabrication process does not require etching of either the silicon material or the YSO crystal.

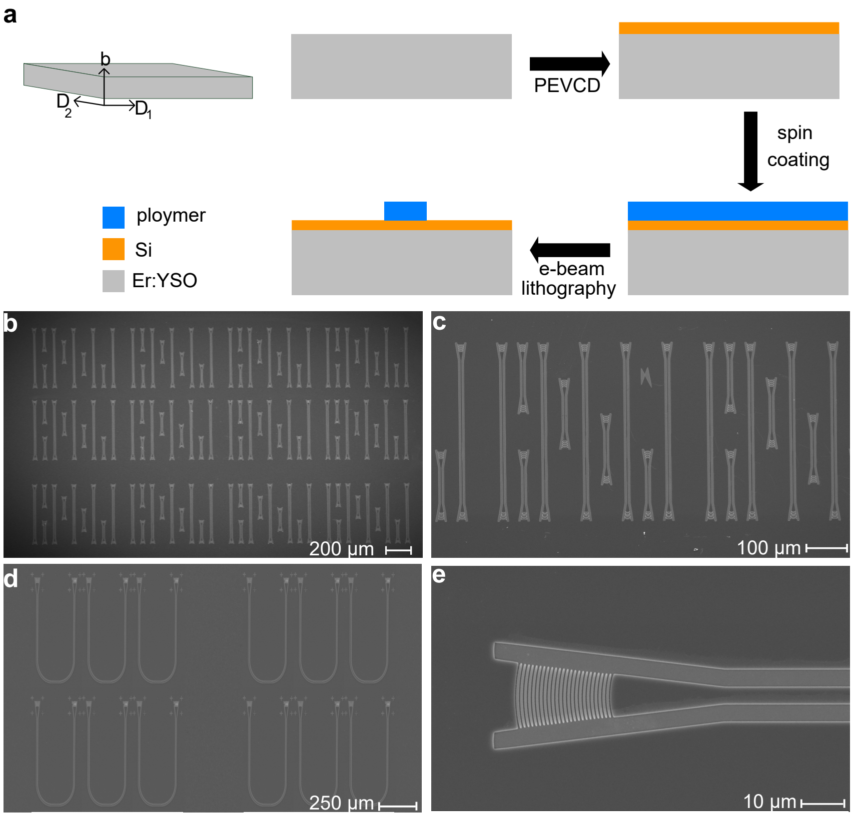


FIG. S1. **Sample fabrications a** Fabrication process. **b** The SEM image of straight waveguides with different widths. **c** Zoomed SEM image of straight waveguides. **d** The SEM image of U-type waveguides with different widths. **e** The SEM image of a grating coupler.

We fabricated a series of straight waveguides and U-shaped waveguides with varying widths and lengths on a single chip. These waveguides were designed to investigate propagation losses

and facilitate echo measurements, respectively. Scanning electron microscope (SEM) images of these structures are presented in Fig. S1b-d.

To determine the propagation loss of the waveguides and identify the Bound States in the Continuum (BIC) condition, ten straight waveguides of identical width were patterned within a 1×1 $mm^2$ region, with widths spanning a range from 0.5 $\mu$m to 5 $\mu$m (Fig. S1b and c). Within each square region, five 850-$\mu$m waveguides and five 250-$\mu$m waveguides, all with the same width, were included for measuring propagation losses (Fig. S1c).

To enhance the interaction between erbium ions and the optical mode, we extended the waveguide length by fabricating U-shaped waveguides, as shown in S1d. Each 1×1 $mm^2$ area incorporates three U-shaped waveguides of identical width, each 1.5 mm long. The U-shaped waveguides feature a 250 $\mu$m separation, enabling efficient light coupling through our 8×1 fiber array and facilitating measurements within a helium cryostat.

Figure S1e shows the SEM image of the grating coupler responsible for light input and output to the chip. Note that all input/output grating couplers for the straight and U-shaped waveguides are identical and have been pre-optimized for optimal performance at 1536-nm wavelength.

## II. DESIGN OF GRATING COUPLER

Figure S2a shows a polarization-sensitive grating coupler used for fiber-to-chip coupling, where light is directed either by a fiber tip (or an output port of a fiber array). The coupling efficiency is affected by the incident angle $\theta$, as illustrated in Fig. S2a. According to diffraction theory, the following equation describes the behavior of grating couplers:

$$\frac{2\pi n_{\text{eff}}}{\lambda}\sin\theta + \frac{2\pi}{\Lambda} = \beta \tag{S1}$$

where $n_{\text{eff}}$ is the effective refractive index of the structure; $\lambda$ is the wavelength of light in vacuum, $\theta$ is the incident angle of light, $\Lambda$ is the period of the grating coupler, $\beta$ is the propagation constant of light in the waveguide.

Figure S2b-d show the results of our numerical simulations using the finite element method (COMSOL Multiphysics). The simulated results indicate that optimal coupling near the erbium ion absorption peak at 1536.47 nm requires a grating period of $\Lambda$ = 0.84 $\mu$m and an incident angle of $\theta$ = 8°.

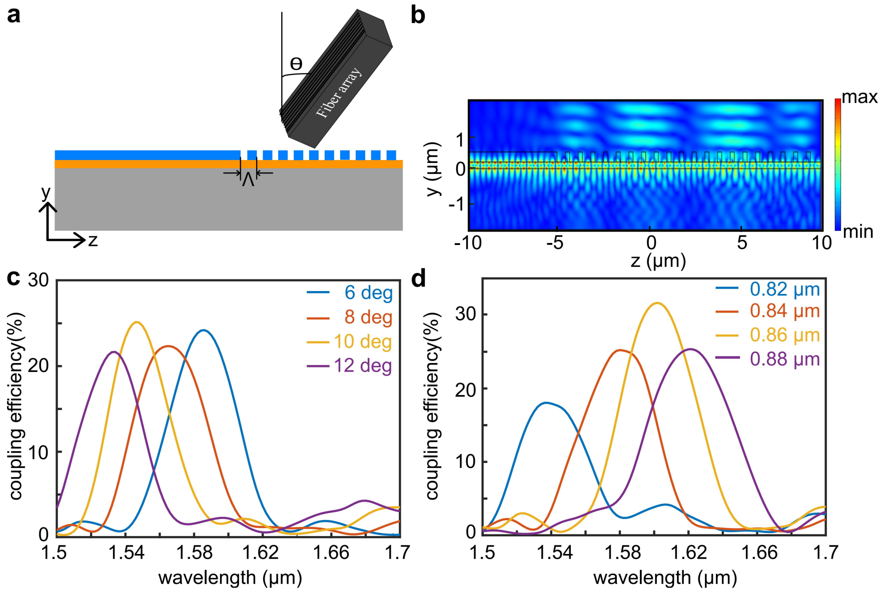


FIG. S2. **Design of the grating coupler.** **a** Illustration of coupling between the grating and waveguide. $\Lambda$, the period of a grating; $\theta$, the incident angle. **b** Electric field distribution illustrating light coupling from a fiber array to the on-chip waveguide. **c** Coupling efficiency of the grating coupler as a function of varying incident angles. The grating period is $\Lambda$ = 0.84 $\mu$m. **d** Coupling efficiency of a grating coupler with varying grating period for a fixed coupling angle of $\theta$ = 8°.

## III. SIMULATIONS OF BIC STRUCTURES

Our BIC optical memory device comprises an organic polymer layer on top of a silicon film, which is deposited on an erbium-doped YSO substrate (Fig. S3**a** and Fig. 1 in the main text). The polymer and silicon layers are 350 and 200 nm thick, respectively, with refractive indices of 1.54 and 3.37, respectively. The biaxial YSO crystal exhibits three optical axes, labeled as **b**, $\mathbf{D}_1$ and $\mathbf{D}_2$, with refractive indices of $n_\mathbf{b} = 1.770$, $n_{\mathbf{D}_1} = 1.769$, and $n_{\mathbf{D}_2} = 1.789$ at the wavelength of 1536 nm [52]. This less than 1% difference in refractive indices was considered negligible in our simulations.

Figure S3a depicts the patterned polymer layer, forming a high-index channel with a width of $w$. The spatial confinement of the polymer within this channel leads to the existence of discrete modes. Beyond the polymer region, both the silicon film and YSO substrate extend infinitely, supporting extended modes with high density of states, i.e., forming a continuum. Our COMSOL simulations confirm that this structure, when the waveguide width is appropriately chosen, supports the coexistence of a transverse-magnetic (TM) bound mode within the continuous spectrum of transverse-electric (TE) modes. Figure S3b illustrates the field distribution of a propagating TE modes within the silicon film. The presence of polymer stripe supports the confinement of the TM mode within the waveguide, as shown in Fig. S3c.

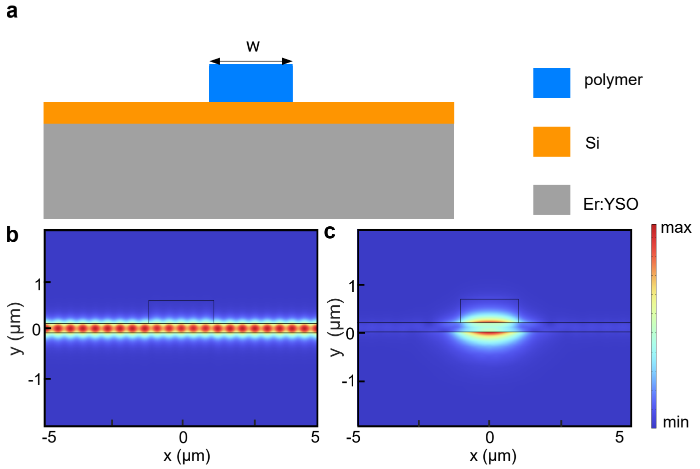


FIG. S3. **Numerical simulation of the BIC waveguide. a** Cross section of the waveguide. **b** Electric field distribution for one of the TE modes supported by the extended boundaries of the silicon layer and the YSO substrate. **c** Electric field distribution for the bound TM mode.

This localization of the TM mode within the TE mode continuum exhibits the characteristic

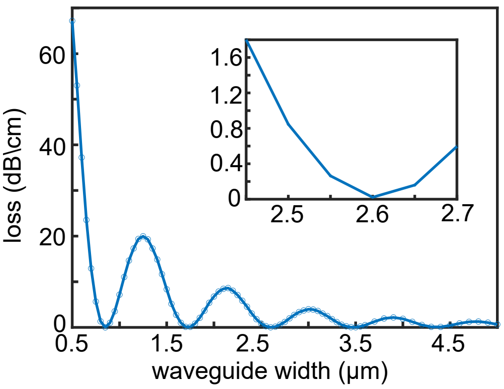


FIG. S4. **Simulated propagation loss of our waveguides as a function of the polymer width** $w$**.** Inset, simulated propagation loss of our waveguides around the polymer width $w$ = 2.6 $\mu$s.

features of a BIC structure, with a near-zero propagation loss and a large portion of the light energy penetrating into the YSO substrate. We numerically simulated the 3D BIC waveguide structure to calculate propagation loss for different waveguide widths. The losses for waveguides of different widths are calculated by integrating the intensity of the light field at the input and output ports. As shown in Fig. S4, at a waveguide width of $w$ = 2.6 $\mu$m, the propagation loss can be as low as 0.02 dB/cm. This propagation loss can be maintained below 1 dB/cm over the waveguide width from 2.5 to 2.7 $\mu$m.

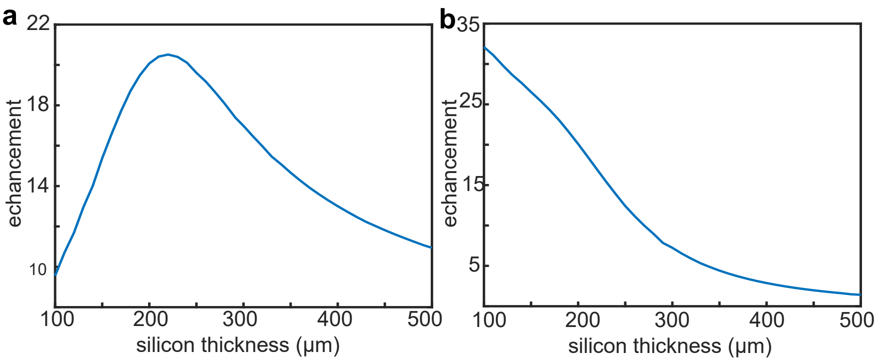


FIG. S5. **Enhanced light absorption of our BIC waveguide.** **a** Absorption enhancement in our BIC waveguide relative to the fundamental TE-like mode in a conventional silicon waveguide as a function of silicon thickness, where the thicknesses in both structures are varied together. **b** Absorption enhancement of our BIC waveguide (variable thickness) compared to the fundamental TE-like mode in a 200 nm $\times$ 600 nm silicon waveguide.

In addition to low propagation loss, our BIC structure also enables a large field penetration into the YSO substrate, indicating a strong interaction with the embedded erbium ions. To quantify this enhanced interaction, we measured the absorption coefficient of an erbium-doped YSO crystal and incorporated this value into simulations of both our BIC waveguides and conventional hybrid structures featuring silicon waveguides. For a silicon waveguide on a YSO substrate with refractive index n=1.78, the TM-like mode in a standard 200-nm-thick silicon waveguide has an effective index of only 1.80. Comperad to the YSO substrate $n = 1.78$; such a low index contrast of TM-like mode is not suitable for guiding light (see Section IV). Therefore, we compared our BIC waveguide to the TE-like mode in conventional silicon waveguides. The simulation result in Fig.S5a shows the absorption enhancement of our BIC waveguide compared to the TE-like mode of a conventional silicon waveguide as a function of silicon thickness, where the silicon thicknesses in both structures are varied together. Figure S5b compares the absorption of our BIC waveguide with varying silicon thickness to a silicon waveguide with fixed dimensions of 600 nm $\times$ 200 nm. These results demonstrate the generality of a notable enhancement in our BIC structures for various waveguide thicknesses. Compared with standard silicon-on-insulator waveguides, the strong absorption demonstrated here signifies an efficient interaction between the light field and erbium ions, which is promising for the development of practical and scalable quantum photonic circuits.

## IV. EXPERIMENTAL MEASUREMENTS OF BIC

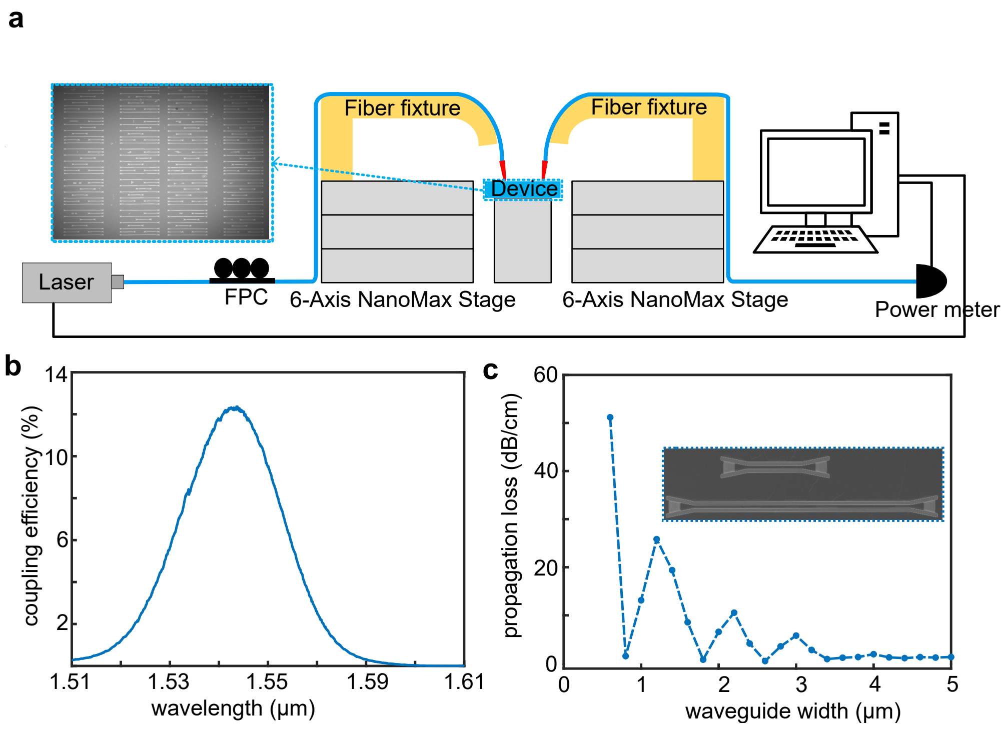


FIG. S6. **Experiment results of the BIC effect. a** Sketch of the experimental setup. FPC, fiber polarization controller; blue lines, fibers; black lines, electric signal lines. Inset, the SEM image of our optical memory device. **b** Coupling efficiency for varying laser wavelength, where the period of a grating coupler is 0.84 $\mu$m and the couping angle is 8°. **c** Measured propagation loss of our waveguide as a function of the waveguide width $w$. Inset, zoomed SEM image of straight waveguide with different lengths to extract the propagation loss of the waveguides.

To experimentally validate the existence of the BIC effect in our structure, we measured the propagation loss for different waveguide lengths and widths (Fig.S6). The experimental setup was illustrated in Fig.S6a. Light from a tunable laser (New Focus TLB-6600) was passed through a fiber polarization controller to adjust its polarization state and then coupled into and out of the optical memory chip via a pair of grating couplers. Two single-mode fibers, mounted on a 6-axis nanopositioning stage, were used to optimize the coupling angle and position between the fibers and the grating couplers. We achieved a maximum coupling efficiency of 13%, as shown in Fig. S6b. The light output from the chip is collected by a single-mode fiber and directed to a power meter.

As mentioned in Sec. I of SM, to investigate propagation loss and identify the BIC condition, we fabricated a series of ten straight waveguides with different widths ranging from 0.5 to 5 $\mu$m, within a 1 mm$^2$ region. Each square region included five waveguides with a length of 850 $\mu$m and five with a length of 250 $\mu$m, all having the same width.

We measured the transmittance of the ten waveguides within a given 1 mm$^2$ region. The propagation loss of straight waveguides was then extracted by comparing the transmittance of the 850 and 250 $\mu$m waveguides. This method allows us to detect a minimal propagation loss of $0.5 \pm 0.5$ dB/cm, which occurs at the waveguide width of $w = 2.6\,\mu$m (Fig. S6c), indicating a BIC point.

## V. MODE PROPERTIES OF CONVENTIONAL SILICON WAVEGUIDES

Decreasing the thickness of a conventional silicon waveguide enhances the optical field overlap with the underlying YSO substrate. This effect is especially pronounced for the TM mode. For example, a silicon waveguide with a cross section of $200 \times 600$ nm$^2$ supports both TE and TM guided modes. The TM mode, in particular, exhibits a distribution with more field confined near the top and bottom surfaces of the silicon layer. However, the effective refractive index of this TM mode is only $n_{\mathrm{eff,TM}} = 1.80$ (Fig.S7a). Compared to the YSO substrate ($n_{\mathrm{YSO}} = 1.78$), this $\sim 1\%$ index contrast presents substantial challenges for waveguide implementation.

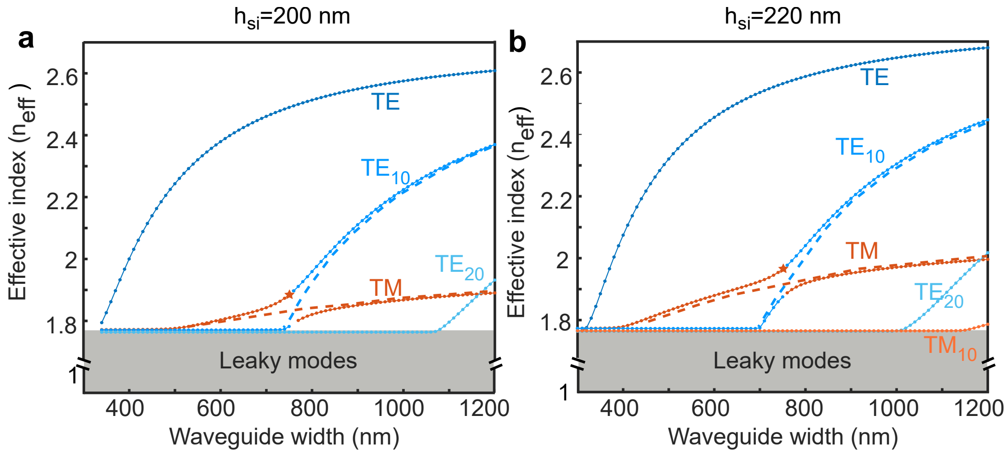


FIG. S7. **Effective indices of silicon waveguides on YSO crystals as a function of waveguide width**. (a) Effective indices of the TE and TM modes for a silicon thickness of 200 nm. (b) Effective indices of the TE and TM modes for a silicon thickness of 220 nm (industrial standard). Color coding: TE modes (blue), TM modes (orange). High order modes such as $TE_{xy}$ are labeled with $x$ and $y$ indicating the mode order in the width and thickness directions, respectively. Stars in both figures indicate points used to plot mode hybridization in Figure S8.

This low index contrast has several detrimental consequences: (1) increased radiation losses at bends, leading to high bending loss; (2) increased sensitivity to fabrication imperfections, resulting in scattering of the weakly confined light out of the waveguide; and (3) increased difficulty in coupling light into and out of the waveguide. As a result, the TM mode is too leaky for reliable light guiding in on-chip optical memories. To quantify this, we employed numerical simulations in two-dimensional waveguides, using the effective index method, to investigate the relationship between bending loss, bending radius, and effective refractive index. Setting the cladding refractive index

to $n$ = 1.78, we vary the effective refractive index of the waveguide to examine the bending loss. The results showed that even at a bending radius of 100 $\mu$ m, a notable bending loss $\sim$ 10 dB/rad persists with an effective refractive index of 1.8. Only when the effective refractive index exceeds 2.1 does the bending loss decrease sufficiently to enable high-density integration on the chip. These results suggest that the TM mode is too leaky to serve as a reliable guiding structure for scalable on-chip optical memories.

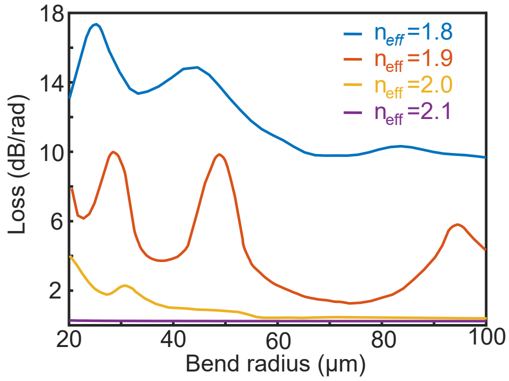


FIG. S8. **Simulation results for bending Loss in 2D simulation** Variation of bending loss with bending radius for different effective indices in traditional silicon waveguide with a waveguide width of 600 nm.

Although increasing the width of the waveguide can, to some extent, increase the effective refractive index, this approach is insufficient to overcome the limitations of the TM mode. As shown in Fig.S7 a, the effective index of the TM mode increases only from 1.80 to 1.90 as the waveguide width is varied from 600 nm to 1200 nm.

Further increasing the waveguide width leads to the emergence of the higher-order TE10 mode. For a waveguide width between 650 nm and 950 nm, the fundamental TM mode hybridizes with the $TE_{10}$ modes, as shown in Fig.S9(a). Increasing the waveguide width beyond 1000 nm results in a transition to the multi-mode regime. As shown in Fig. S10, the waveguide becomes highly sensitive to thickness variations at this width, with several higher-order modes arising across a wide range of thicknesses. This mode hybridization and multi-mode behavior complicates the control and manipulation of light, and may cause reduced fidelity of opitcal signals.

Standard industrial silicon chips typically have a 220 nm thickness, slightly higher than the 200 nm used above. However, the problems of low effective index, mode hybridization, and multi-mode operation persist. The TM mode's effective index for 220-nm-thick silicon increases only from 1.87 to 2.00 as the waveguide width is varied from 600 nm to 1200 nm (Fig.S7 b). Within the

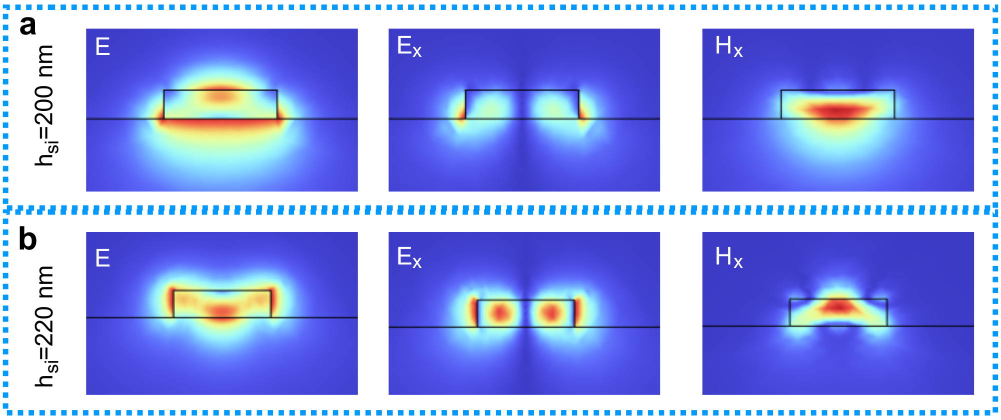


FIG. S9. **Mode hybridization of in silicon waveguide.** **a** Field distribution of the hybrid mode in a 200×720 nm$^2$ silicon waveguide, as indicated by the star in Figure S7(a). **b** Field distribution of the hybrid mode in a 220×750 nm$^2$ silicon waveguide, as indicated by the star in Figure S7(b).

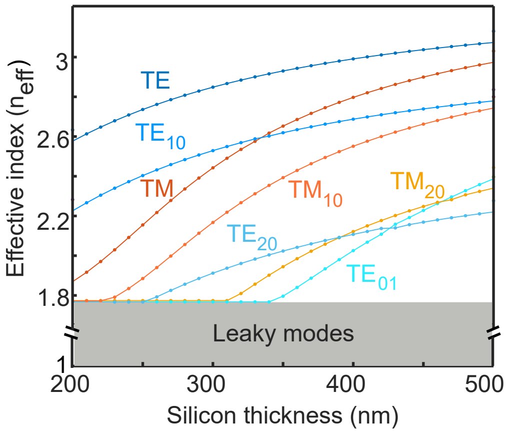


FIG. S10. **Effective indices of silicon waveguides on YSO crystals as a function of silicon thickness**. The waveguide width is 1000 nm. High order modes such as $TE_{xy}$ are labeled with $x$ and $y$ indicating the mode order in the width and thickness directions, respectively.

waveguide width range of 550 to 900 nm, the propagating modes manifest themselves as hybrid modes, combining the features of the fundamental TM mode and a higher-order transverse $TE_{10}$ mode, as shown in Fig.S9b, S10 b.

## VI. EXPERIMENTAL SETUP AT HELIUM TEMPERATURE

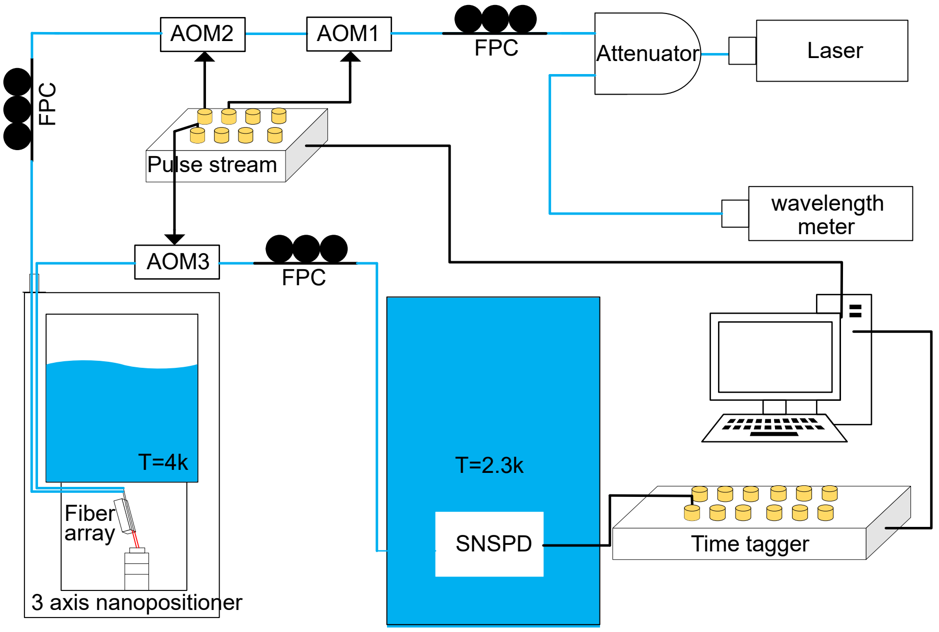


FIG. S11. **Sketch of the experimental setup at helium temperature.** The attenuator is a 95:5 fiber optic attenuator. The pulse stream is used to generate synchronous digital patterns. The time tagger is employed for streaming time-to-digital converters. AOM, acousto-optic modulator; FPC, fiber polarization controller; SNSPD, superconducting nanowire single-photon detector. Blue lines, fibers; black lines, electric signal lines.

The experimental setup, illustrated in Fig. S11, is composed of four distinct components: a laser source, a pulse control system, a cryostat for optical chip testing, and a single-photon superconduction nanowire detector (SNSPD).

In our helium-temperature optical measurements, we used a tunable fiber laser (Koheras Adjustik E15) with a linewidth less than 100 Hz. This laser beam was split into two paths: 5% of the light was directed to a wavelength meter (Bristol 671A) for accurate frequency measurement (MHz resolution). The remaining 95% of the light was transmitted, enabling the adjustment of light intensity through additional approaches.

This light then traveled through a fiber polarization controller (FPC) to control its polarization before being modulated by two acousto-optic modulators (AOM1 and AOM2) to generate time-sequence pulses. The combination of the two AOMs can achieve an on-off extinction ratio exceeding 80 dB.

After passing through another FPC for polarization control, the laser pulses were coupled into the memory chip via a fiber-coupled array with 8 x 1 outputs. The memory chip was mounted on a gold-coated copper plate, which was positioned using a 3-axis nanopositioner and placed within a home-built helium cryostat, cooled to 4 K.

The output signal of the memory chip was collected by the same fiber array, passed through another acousto-optic modulator (AOM3) and a fiber polarization controller (FPC), and directed to a SNSPD operating at 2.3 K. AOM3 was introduced to control the timing of SNSPD detection. The SNSPD signal is read out using a time tagger (Swabian Instruments TT20). The AOMs are controlled by a pulse stream generator (Swabian Instruments Pulse Streamer 8/2). The pulse stream generator, the time tagger, and all the AOMs are synchronized with precise control over delay by a computer, ensuring accurate timing for the experiment.

## VII. TIME SEQUENCES OF LASER PULSES

AOM1 and AOM2 are used to generate the specific pulse sequence needed to interact with the optical chip and to notablely reduce the on-off extinction ratio of the overall system. AOM3 is employed to control the precise timing of the SNSPD detection window.

For the photoluminescence (PL) intensity measurement, as shown in Fig. S12**a**, the excitation pulse has a duration of 20 ms and the fluorescence collection window is 50 ms. The system then waits 100 ms before repeating the pulse sequence.

For photon-echo mearsurement, as shown in Fig. S12**c**, the duration of first pulse and second pulse are 0.31 $\mu$s and 0.62 $\mu$s, respectively. The wait time between two pulses is $\tau$. The system then waits 100 ms before repeating the pulse sequence.

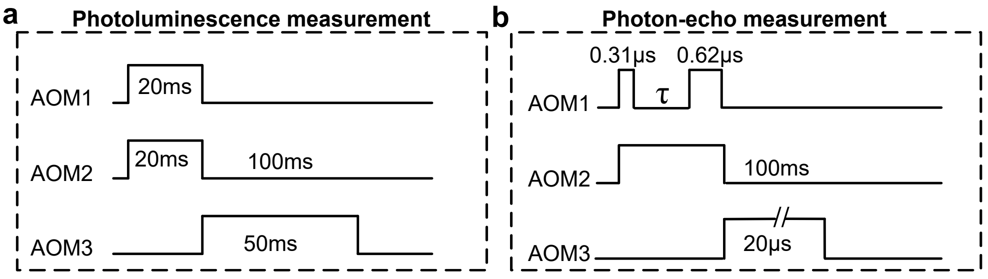


FIG. S12. **Time sequences of applied laser pulses.** **a** Time sequence for photoluminescence measurement. **b** Time sequence for photon-echo measurement.

## VIII. PHOTOLUMINESCENCE MEASUREMENT

To confirm that the optical field interacts with the erbium ions as expected, we applied a 20-ms resonant excitation pulse to the erbium ions in the memory chip and then measured their PL across a range of frequencies. The results of this measurement, presented in Fig. S13**a**, demonstrate that the erbium ions consistently exhibit a lifetime of 11.5 ms across different frequencies.

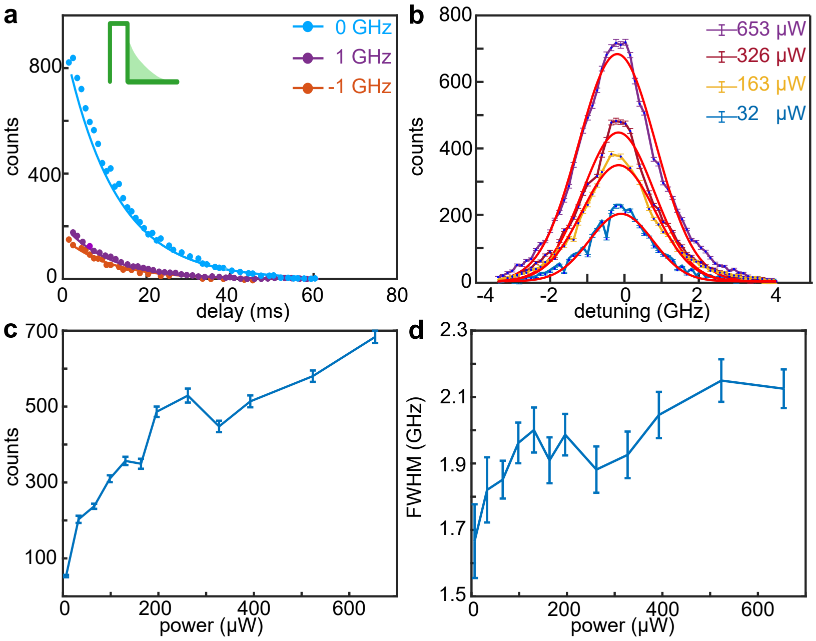


FIG. S13. **Coupling between erbium ions and the BIC waveguide. a** Photoluminescence intensity of erbium ions under resonant excitation with a 20 ms pulse, measured at various laser frequencies as noted. The integration time of the SNSPD was set to 50 ms. Points, experimental points; solid lines, fitted curves. **b** Peak counts of the PL signal for different laser detunings, measured at varying laser powers as noted. Points, experimental points; solid lines, fitted curves. **c** PL peak counts as a function of input laser power, where the laser frequency was set to 0 GHz. **d** Full width at half maximum (FWHM) of the fitted curves in **b** as a function of laser power. The FWHM increases from 1.6 GHz at low excitation power to 2.1 GHz at high power.

PL signal from erbium ions was observed over a wide range of laser frequencies, as shown in Fig. S13**b**. Despite an overall increase in peak photon count with increasing laser power, these increases exhibited oscillations (Fig. S13**c**). By fitting the data with a Lorentzian function (solid curves in Fig. S13**b**), we found that the inhomogeneous linewidth increases from 1.6 GHz at a low excitation power of 32 $\mu$W to 2.1 GHz at a high power of 653 $\mu$W. This broadening is attributed

to the long excited-state lifetime of erbium ions ($T_1$ = 11 ms). With an electric-dipole moment of approximately $2 \times 10^{-30}$ C·m for the erbium ions and the BIC waveguide cross section of $8 \times 10^{-13}$ $m^2$ (simulated result), an input laser power on the order of 10 $\mu$W into the chip results in a Rabi frequency $\Omega \sim$ MHz. The saturation linewidth, theoretically proportional to the square of the Rabi frequency ($\Omega^2$) and the excited state lifetime $T_1$, leads to the saturation of the inhomogeneous GHz linewidth of erbium ions. When the input power exceeds 400 $\mu$W, the linewidth of the PL signal remains relatively constant, indicating that the entire inhomogeneous line of the erbium ions is saturated, as shown in Fig. S13**d**.

The use of time-resolved detection for our photon echo measurements is ideal. However, our current experimental setup faces limitations when attempting time-resolution below 1 $\mu$s. First, the high data acquisition rate of our single-photon counting system leads to overflow issues in our system's memory. Second, such measurements with shorter resolution time require longer averaging time for reasonable signal-to-noise ratios. The output of our laser is subject to polarization change, and therefore, the these fluctuations would require long-term stabilization, making it hard to achieve with our current setup.

## IX. MEASUREMENTS OF REFERENCE BULK MATERIAL

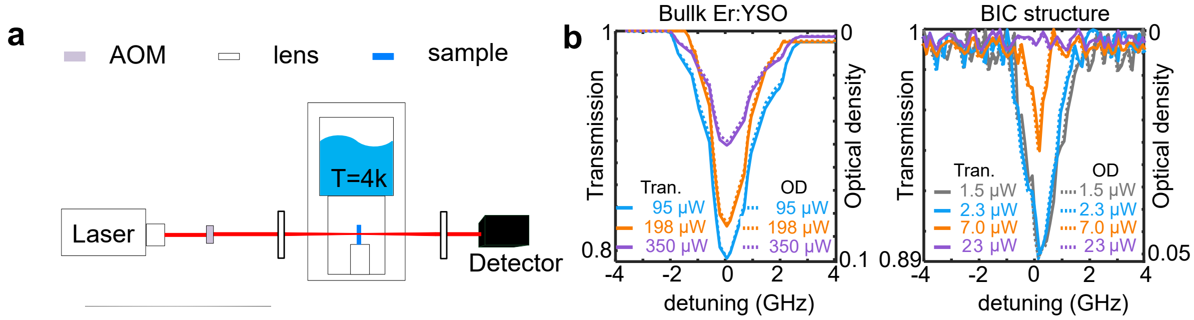


FIG. S14. **Experimental demonstration of the bulk Er:YSO. a** Sketch of the experimental setup for a bulk material. AOM, acousto-optic modulator. White rectangle, $f = 300\,\mathbf{mm}$ optical lens. **b** Absorption our bulk crystal with varying laser power. Solid lines, transmission spectra; dashed lines, optical density curves. **d** Absorption spectra for varying laser power. Left, bulk material; right, BIC devices. Solid lines, transmission spectra; dashed lines, optical density curves.

The absorption coefficient of our BIC waveguide is consistent with its BIC properties, which indicates that 45% of the optical field is confined within the YSO crystal (Fig. 2 in the main text).

To provide a comparative reference, optical measurements were performed on a bulk Er:YSO sample with the same doping concentration of 38 ppm. The experimental setup is illustrated in Fig. S14**a**. The bulk Er:YSO sample was placed in our Helium cryostat and cooled down to 4 K. Light from a tunable fiber laser, polarized along the $\mathbf{D_1}$ axis of the YSO crystal, was directed onto the sample. The transmitted light was then collected by a lens and directed to a detector.

The left panel of fig. S14b shows the absorption of the bulk material for different input laser powers. By sweeping the laser frequency, we measured the inhomogeneous absorption linewidth for varying laser powers. We observe saturation of the inhomogeneous erbium linewidth when the input power exceeds 200 $\mu$W. At low input laser powers, the measured inhomogeneous linewidth is 1.6 GHz with a peak absorption depth of 21%, which corresponds to an absorption coefficient of 2.5 cm$^{-1}$.

The length of our BIC waveguide determines the optical depth available to absorb the input signal pulse. Due to the optimal scan area of our electron beam lithography system, the maximum length we can fabricate is 1.5 mm, resulting in an absorption of 11.5% of the input light, as shown in The right panel of fig. S14b. This corresponds to an absorption coefficient of 0.8 cm$^{-1}$, approximately 32% of the absorption coefficient of the bulk Er:YSO (2.5 cm$^{-1}$).

While our simulations indicate that 45% of the electric field within the BIC structure interacts with the YSO crystal, the observed absorption of 32% is slightly lower than this prediction. This discrepancy is attributed to the polarization dependence of erbium ion absorption, which exhibits stronger absorption when the polarization aligns with the $\mathbf{D_1}$ axis of the YSO crystal. The confined optical field of the BIC mode has field components along the **b**, $\mathbf{D_1}$ and $\mathbf{D_2}$ axes, resulting in a reduced absorption of 32% compared to the predicted 45%.

To further characterize the absorption linewidth under different laser powers,we converted our transmission data of the BIC structure and bulk material to Optical density (OD) as shown by the dashed line in fig.S14 b. Under varying power conditions, the inhomogeneous linewidths of the OD from both the bulk material and BIC structure show a consistency with the inhomogeneous absorption linewidth. Notably, at low power, both the bulk material and BIC structure exhibited inhomogeneous linewidths of approximately 1.6 GHz. These findings provide additional support for the conclusion that the BIC structure effectively conserves the inhomogeneous linewidth of the bulk material.

## X. HOMOGENEITY OF THE MEMORY CHIP

Our experimental investigations involving multiple waveguides across a $4 \times 4$ mm$^2$ area on a single chip revealed consistent opitcal properties of erbium ions, which confirms the homogeneity of the BIC device.

We fabricated several U-type waveguides with varying widths within $4 \times 4$ mm$^2$ area on the same chip, as shown in Fig.S1. To show the homogeneity of our device, we measured the photoluminescence (PL) of erbium ions in multiple waveguides, as presented in Fig. S15. The PL signal remains consistent among them as the excitation pulse power increases over a range exceeding one order of magnitude.

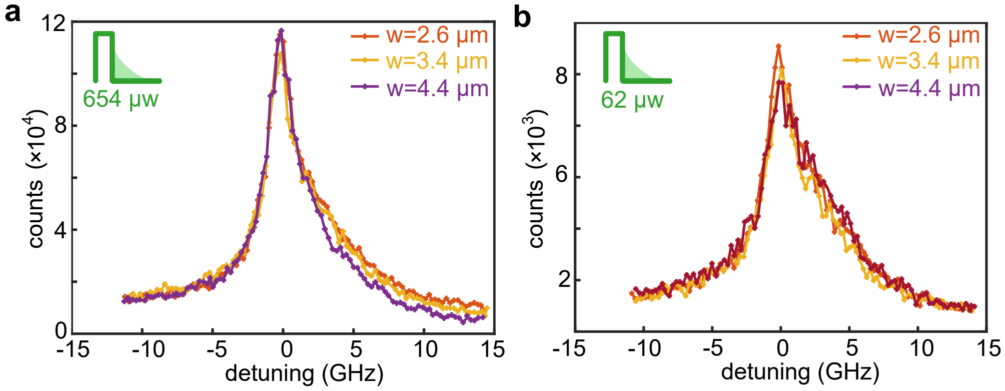


FIG. S15. **Photoluminescence of different devices.** **a** Photoluminescence spectrum measured a resonant pulsed excitation for 654 $\mu$W. **b** Photoluminescence spectrum measured a resonant pulsed excitation for 62 $\mu$W. The excitation pulse has a duration of 20 ms.
